# Burnout, resilience, and cardiometabolic phenotype in an academic medical faculty: an integrated cross-sectional assessment from the United Arab Emirates

**DOI:** 10.3389/fendo.2026.1913703

**Published:** 2026-08-17

**Authors:** Adnan Agha, Javed Yasin Pathan, Eram Anwar, Hayat Eltayeb, Mussab Fadl Elseed, Ahmed Suhail Ashraf Ali, Juma Al Kaabi

**Affiliations:** 1 Department of Internal Medicine, College of Medicine and Health Sciences, United Arab Emirates University, Al Ain, United Arab Emirates; 2 Clinical Trials Unit, College of Medicine and Health Sciences, United Arab Emirates University, Al Ain, United Arab Emirates

**Keywords:** burnout, medical faculty, metabolic dysfunction-associated steatotic liver disease, resilience, United Arab Emirates (UAE)

## Abstract

Cardiometabolic risk in the Gulf academic medical faculty remains poorly characterised, and whether this co-clusters with occupational burnout has not been examined in any published Gulf cohort. We combined objective cardiometabolic phenotyping with structured burnout and resilience scoring in a single faculty cohort during a 1-week screening camp in November 2025, enrolling 110 faculty members of the College of Medicine and Health Sciences, United Arab Emirates University. Burnout was assessed with the Maslach Burnout Inventory–Human Services Survey for Medical Personnel (MBI-HSS-MP) and resilience with the 10-item Connor-Davidson Resilience Scale (CD-RISC-10). Each participant underwent anthropometry, two blood pressure readings, fasting biochemistry, transient elastography with controlled attenuation parameter (CAP) and liver stiffness measurement (LSM), common carotid intima–media thickness assessment, and Framingham 10-year risk computation. Group differences were tested using the Mann–Whitney *U* test, correlations with Spearman’s *ρ*, and adjusted associations with linear or logistic regression (covariates: age, sex, body mass index; liver outcomes additionally adjusted for glycated haemoglobin). Reporting followed STROBE guidelines. The MBI-complete analytic cohort numbered 106 (median age 43.0 years; 50.9% men). Any-dimension burnout under the 4th-edition criteria affected 65 of 106 (61.3%) faculty. The cohort showed broad cardiometabolic risk: overweight 32.1%, obesity 26.4%, hypertension 15.1%, prediabetes 16.5%, elevated triglyceride–glucose index 51.5%, elevated atherogenic index of plasma 24.3%, high lipoprotein(a) ≥125 nmol/L in 17.5%, and metabolic dysfunction-associated steatotic liver disease in 55.9% of 102 FibroScan-tested participants. After adjustment, emotional exhaustion was independently associated with LSM (*β* = 0.061 kPa per point; 95% CI 0.022 to 0.100; *P* = 0.002), as was depersonalisation (*β* = 0.106 kPa per point; 95% CI 0.025 to 0.187; *P* = 0.011); the latter was also associated with CAP (*β* = 2.43 dB/m per point; 95% CI 0.51 to 4.35; *P* = 0.014). Resilience and emotional exhaustion were inversely correlated (*ρ* = −0.27, *P* = 0.006), whereas resilience and personal accomplishment were positively correlated (*ρ* = 0.31, *P* = 0.001). Cardiometabolic risk and burnout were both common and overlapped along specific dimensions rather than uniformly, with emotional exhaustion and depersonalisation tracking measurable liver phenotype. Routine cardiometabolic and hepatic phenotyping should accompany dimension-specific burnout screening in faculty wellness pathways across the Gulf.

## 1 Introduction

Cardiovascular disease is the leading cause of non-communicable mortality in the United Arab Emirates, accounting for approximately 34% of all deaths and producing a modelled prevalent cohort exceeding 700,000 cases nationally (1, 2). Risk factor accumulation begins early in this population, with national STEPS data showing that nearly half of UAE adults aged 18 to 44 years carry three or more cardiovascular risk factors and population-screening cohorts reporting obesity in 35%, diabetes in 18%, dyslipidaemia in 44%, and hypertension in 23% of Emirati adults (1, 3, 4). Atherogenic dyslipidaemia, central adiposity, and metabolic dysfunction-associated steatotic liver disease (MASLD) are particularly common in Gulf cohorts and are increasingly recognised as drivers of premature cardiovascular events in middle-aged adults of South Asian and Middle Eastern backgrounds (5–7).

Faculty in academic medicine occupy an awkward position in this risk landscape. Clinical service, teaching, and competitive research demands stack up against a working culture that rarely prioritises personal health. Burnout syndrome, characteristically identified by Maslach’s three dimensions of emotional exhaustion, depersonalisation, and reduced personal accomplishment, has been documented across diverse physician and healthcare worker cohorts, often above 40% (8, 9). Beyond the psychological footprint, mechanistic work has tied burnout to cortisol dysregulation, blunted interleukin-6 reactivity, coronary microvascular dysfunction, and adverse metabolic phenotypes (9–12). The clinical signal across cohorts is heterogeneous. A Tunisian university hospital conference reported an association between burnout and endocrine/metabolic disorders in healthcare workers (13); a Taiwanese tertiary hospital study showed an adjusted association between burnout and metabolic syndrome amongst doctors and nurses (14); and a UAE-based study reported a negative association between cardiovascular health (Fuster-BEWAT) and emotional exhaustion during the COVID-19 pandemic (15). These differences reflect variations in instrument versions, case definitions, and cohorts, and not a settled question.

Resilience is the personal capacity to adapt to adversity and is increasingly conceptualised as a protective construct that buffers the impact of occupational stress on burnout and downstream health outcomes (16, 17). Inverse associations between resilience and burnout have been demonstrated in nursing and physician samples; however, resilience data from Middle Eastern academic medical faculty are sparse, with the most comprehensive Gulf and South Asian healthcare worker resilience evidence drawn primarily from Iranian and Saudi nursing cohorts using the 25- or 10-item Connor-Davidson Resilience Scale (16–18).

Three evidence gaps follow from this literature. First, to our knowledge, no published study has integrated objective cardiometabolic phenotyping with structured burnout and resilience measurements in an academic medical faculty in a Gulf setting. Second, prior studies have generally relied on self-reported cardiometabolic outcomes or a limited biomarker panel, without transient elastography for hepatic steatosis and stiffness, common carotid intima–media thickness, or surrogate atherogenic indices, including the triglyceride–glucose index, atherogenic index of plasma, Castelli risk indices, lipid accumulation product, and visceral adiposity index (13–15). Third, no study has examined whether the three MBI-HSS-MP burnout dimensions differ in their associations with measurable cardiometabolic phenotypes, despite mechanistic reasons to expect that emotional exhaustion (the load dimension) and depersonalisation (the disengagement dimension) may have different physiological correlates than reduced personal accomplishment.

We addressed these gaps by conducting an integrated cross-sectional assessment of cardiometabolic risk, burnout, and resilience in a faculty at the College of Medicine and Health Sciences (CMHS) at the United Arab Emirates University. The primary objective was to characterise the prevalence of any-dimension burnout, latent burnout profiles, and measurable cardiometabolic phenotypes in this cohort. The secondary objective was to examine whether burnout (overall and by dimension) was associated with hepatic steatosis, liver stiffness, atherogenic dyslipidaemia surrogates, and the Framingham 10-year cardiovascular risk after adjusting for age, sex, and body mass index. We hypothesised that emotional exhaustion and depersonalisation would be more closely associated with measurable hepatic and lipid phenotypes than reduced personal accomplishment and that resilience would be inversely related to burnout dimensions in this academic medical population.

## 2 Patients and methods

### 2.1 Study design and setting

We conducted a single-centre cross-sectional study during a 1-week faculty health screening camp at the College of Medicine and Health Sciences (CMHS), United Arab Emirates University, Al Ain, in November 2025. Ethics approval was granted by the UAEU Human Ethics Committee (reference ERH_2024_5461), and all participants provided written informed consent before data collection. We followed the Strengthening the Reporting of Observational Studies in Epidemiology (STROBE) statement for cross-sectional studies (19).

### 2.2 Participants

**Figure 1:**
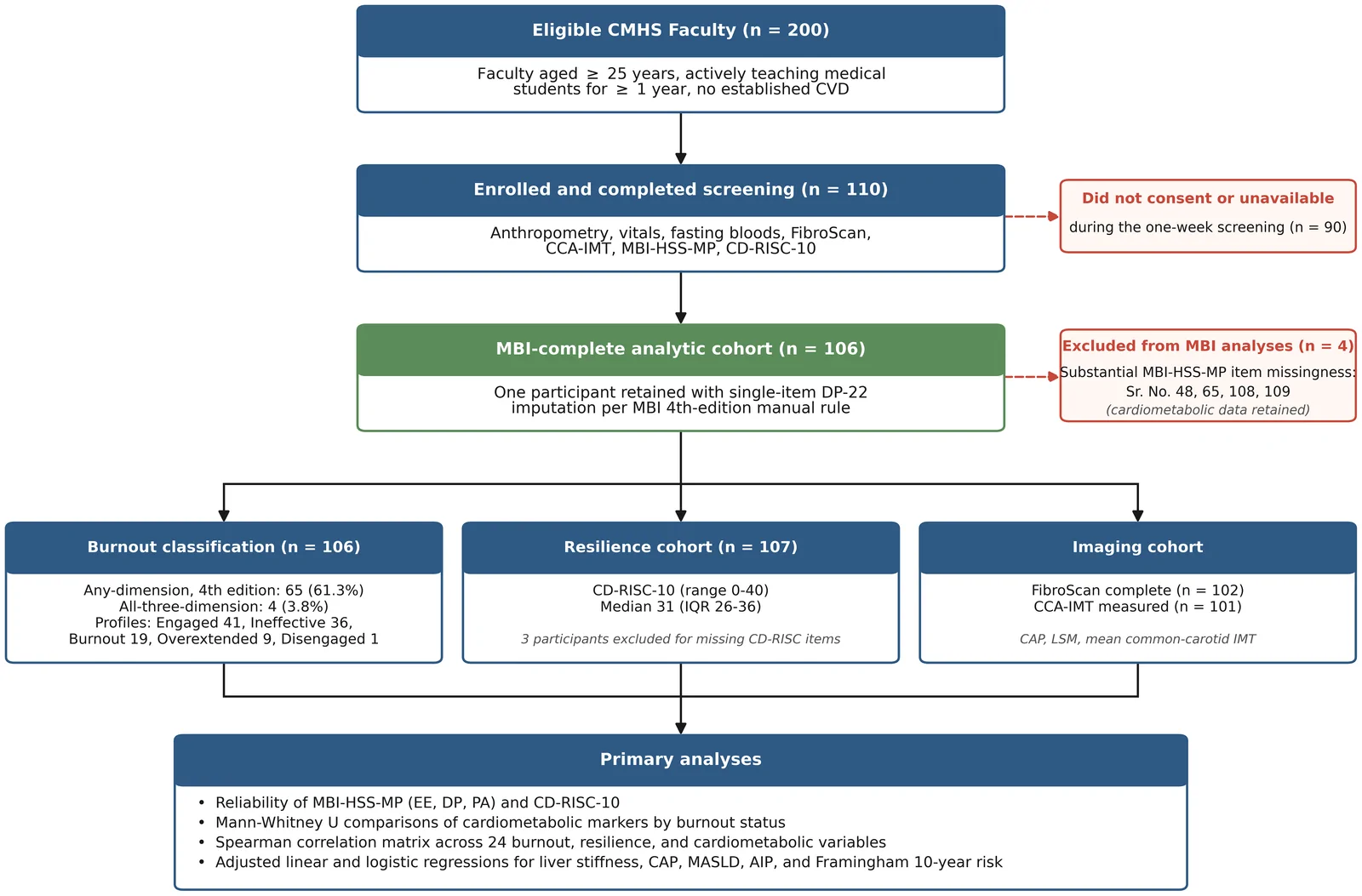
STROBE participant flow diagram. Recruitment, eligibility, MBI-HSS-MP completion, and cohort splits (set A versus set B) for the College of Medicine and Health Sciences (CMHS) faculty cardiometabolic, burnout, and resilience study, November 2025. Reporting follows the Strengthening the Reporting of Observational Studies in Epidemiology (STROBE) statement. Four participants were excluded from MBI analyses for substantial item missingness; one participant on maintenance haemodialysis was identified *post hoc* and excluded from biochemistry-based and Framingham analyses (set B cohort *n* = 105; *n* = 103 with assayed biochemistry for descriptive prevalence; *n* = 104 for set B regression after complete-case covariate filtering. FibroScan-complete cohort *n* = 102; common carotid intima–media thickness (CCA-IMT) available for 101 of 106 MBI-complete participants. MBI-HSS-MP, Maslach Burnout Inventory–Human Services Survey for Medical Personnel; CD-RISC-10, 10-item Connor-Davidson Resilience Scale; CAP, controlled attenuation parameter; LSM, liver stiffness measurement; MASLD, metabolic dysfunction-associated steatotic liver disease; AIP, atherogenic index of plasma.

Eligible participants were CMHS faculty members aged ≥25 years who were actively engaged in undergraduate or postgraduate medical student teaching for at least 1 year. Exclusion criteria were established cardiovascular disease at enrolment, defined as prior myocardial infarction, percutaneous coronary intervention, coronary artery bypass grafting, ischaemic stroke, transient ischaemic attack, or peripheral arterial disease. Pregnant participants were eligible for enrolment but were excluded from FibroScan and Framingham score calculations; no pregnant participants were enrolled in this study. From an estimated eligible pool of approximately 200 faculty, 110 participants completed enrolment, corresponding to an estimated participation rate of approximately 53%. Recruitment, eligibility assessment, and analytic exclusions are illustrated in **Figure 1**.

One participant with established end-stage renal failure on maintenance haemodialysis was identified *post hoc* through an outlying serum creatinine value (990 µmol/L) and an estimated glomerular filtration rate (eGFR) of approximately 5 mL/min/1.73 m². This participant was retained for psychometric (MBI, CD-RISC-10), anthropometric, blood pressure, and FibroScan analyses but was excluded from all biochemistry-based analyses (lipid panel, atherogenic surrogate indices, Framingham 10-year cardiovascular risk score, and eGFR-stratified sensitivity) because the lipid and inflammatory profiles in this participant reflected pharmacotherapy and renal replacement therapy effects rather than a physiological cardiometabolic phenotype. Throughout the manuscript, two analytic cohorts were used: set A (the MBI-complete cohort, *n* = 106, with the haemodialysis participant retained) for psychometric, anthropometric, blood pressure, and FibroScan analyses; and set B (the biochemistry-derived cohort, *n* = 105 after exclusion of the haemodialysis participant) for descriptive lipid, atherogenic surrogate, and Framingham analyses. Two further set B participants had no blood sample assayed; descriptive prevalence estimates in the results therefore use the available measurement denominator of *n* = 103 within set B (the convention is stated in Section 2.7).

### 2.3 Anthropometry, vital signs, and biochemistry

Standing height was measured to the nearest 0.1 cm and body weight to the nearest 0.1 kg using a calibrated stadiometer and electronic scale. Body mass index (BMI) was calculated as weight in kilograms divided by height in metres squared. Waist and hip circumferences were measured at the level of the iliac crest and the greatest gluteal protrusion, respectively, and the waist-to-hip ratio (WHR) was derived. Brachial blood pressure was measured twice with the participant seated quietly for at least 5 min using a calibrated automated oscillometric device, and the mean of the two readings was recorded. Prehypertension was defined as systolic blood pressure 120–139 mmHg or diastolic blood pressure 80–89 mmHg in the absence of antihypertensive treatment, and hypertension was defined as systolic blood pressure ≥140 mmHg, diastolic blood pressure ≥90 mmHg, or current antihypertensive use. Fasting venous blood was drawn after at least 8 h of fasting and analysed in the UAEU clinical laboratory for fasting glucose, glycated haemoglobin (HbA1c), total cholesterol, low-density lipoprotein cholesterol (LDL-C), high-density lipoprotein cholesterol (HDL-C), triglycerides, lipoprotein(a) (Lp(a)), high-sensitivity C-reactive protein (hs-CRP), alanine and aspartate aminotransferase, gamma-glutamyl transferase, alkaline phosphatase, albumin, total protein, and 25-hydroxyvitamin D. Lp(a) values reported by the laboratory as exceeding the assay’s upper limit of quantification (>240 nmol/L) were recoded as censored-high and counted within the ≥125 nmol/L high-Lp(a) category for categorical analyses; they were excluded from continuous distribution summaries and from any computation requiring a point estimate (e.g., Spearman correlations involving Lp(a) as a continuous variable).

### 2.4 Cardiometabolic surrogate indices and risk score

Five smoking-independent atherogenic surrogate indices were calculated from the lipid and anthropometric data. The triglyceride–glucose index (TyG) was computed as ln([triglycerides in mg/dL × fasting glucose in mg/dL]/2), with values exceeding 8.6 considered elevated (20). The atherogenic index of plasma (AIP) was computed as log10(triglycerides/HDL-C in mmol/L), with values above 0.21 considered elevated (21). Castelli risk indices I and II were computed as total cholesterol divided by HDL-C and as LDL-C divided by HDL-C, respectively, with Castelli I of ≥4.5 considered elevated. The lipid accumulation product (LAP) was computed as [(waist circumference in cm − 65) × triglycerides] for men and [(waist circumference − 58) × triglycerides] for women. The visceral adiposity index (VAI) was computed using standard published formulae for sex-specific waist, BMI, triglyceride, and HDL-C inputs (22). eGFR was calculated using the Chronic Kidney Disease Epidemiology Collaboration (CKD-EPI) 2021 race-free equation (23).

The Framingham 10-year general cardiovascular risk (FRS) was derived using the lipid-based equation of D’Agostino et al. (24). Because the screening protocol did not capture smoking history at the visit, we substituted sex-stratified national probabilities—0.15 for men and 0.02 for women—drawn from UAE population data. Each participant therefore carries a probability-weighted Framingham value bracketed by two sensitivity bounds: a non-smoker-equivalent estimate (smoking = 0) and a smoker-equivalent estimate (smoking = 1). The primary value should be read as a population-mean expected-risk profile, not as an individual prediction. MASLD was defined per Eddowes et al. (25, 26) as a transient-elastography CAP ≥238 dB/m and advanced fibrosis as LSM ≥9.7 kPa (F3 or higher). Prevalence of MASLD and advanced fibrosis is reported using the FibroScan-tested denominator (*n* = 102) throughout.

### 2.5 Liver FibroScan and carotid imaging

Vibration-controlled transient elastography was performed by a trained operator using a FibroScan device (Echosens, Paris, France) with the appropriate M or XL probe selected by an automated probe-selection tool. Both LSM (in kPa) and CAP (in dB/m) were recorded as the median of at least 10 valid measurements with an interquartile range to median ratio of <30%. Steatosis severity was graded by CAP (S0 <238, S1–238 to 259, S2–260 to 289, and S3 ≥290 dB/m) and fibrosis stage by LSM (F0–F1 <7.0, F2 7.0 to 9.6, F3 9.7 to 13.5, and F4 ≥13.6 kPa) (25). Common carotid artery intima-media thickness (CCA-IMT) was measured 1 cm proximal to the carotid bulb on B-mode ultrasound using semiautomated edge-detection software, with the mean of the left and right common carotid artery measurements recorded as the participant CCA-IMT. CCA-IMT data were available for 101 of the 106 MBI-complete participants.

### 2.6 Burnout and resilience instruments

Burnout was measured using the 22-item Maslach Burnout Inventory–Human Services Survey for Medical Personnel (MBI-HSS-MP), licenced from Mind Garden Inc., which generates three subscale scores: emotional exhaustion (EE; nine items, range 0–54), depersonalisation (DP; five items, range 0–30), and personal accomplishment (PA; eight items, range 0–48), each rated from 0 (never) to 6 (every day) (27). Burnout classification used the MBI 4th-edition sample-derived *z*-score profile cutoffs (high EE > mean + 0.5 SD, high DP > mean + 1.25 SD, low PA < mean + 0.10 SD) and the latent burnout profile framework (Engaged, Ineffective, Overextended, Disengaged, Burnout) as per Leiter and Maslach (27, 28). The 3rd-edition normative tertile cutoffs were not used because they were withdrawn from the MBI Manual 4th edition by Maslach et al. on the grounds of insufficient diagnostic validity (27, 28).

Resilience was measured using the 10-item Connor-Davidson Resilience Scale (CD-RISC-10), with each item rated on a scale of 0 (not true at all) to 4 (true nearly all the time), and the total score range was 0–40 (29, 30). Higher scores indicate greater resilience. Both instruments were administered in English, the language of medical education at CMHS.

### 2.7 Sample size and statistical analysis

Because the source population was small (fewer than 200 eligible CMHS faculty), the study was framed as a high-coverage faculty health-screening cohort, not a conventional population-sampling study. Under the conservative assumption of a 200-faculty source, the achieved MBI-complete sample of 106 corresponds to at least 53.0% coverage. Applying the finite-population correction (factor 0.687 at *P* = 0.5) shrinks the prevalence 95% margin of error to ±6.5 percentage points, compared with ±9.5 points without correction. With *n* = 106, the study had at least 80% power at two-sided *α* = 0.05 to detect a Spearman correlation of |*r*| ≥ 0.27; with group sizes of 65 versus 41, at least 80% power to detect a between-group standardised mean difference *d* ≥ 0.56. For binary outcomes at a 50% baseline event probability, the minimally detectable odds ratio at 80% power was 3.26; therefore, the logistic analyses were powered for large rather than moderate effects. For adjusted linear regression with four to six covariates, the minimally detectable incremental effect was partial *f*² ≈ 0.078 (incremental partial *R*² ≈ 7.2% to 7.3%).

Continuous variables were tested for normality using the Shapiro–Wilk test and reported as mean (SD) when approximately normal, otherwise as median (IQR). Categorical variables are reported as frequencies (percentages). Between-group testing used the Mann–Whitney *U* or independent-sample *t*-test for continuous variables and the chi-square or Fisher’s exact test for categorical variables, with Spearman’s *ρ* for bivariate associations. Linear regression was applied to the LSM, CAP, AIP, Castelli I, LAP, and Framingham 10-year risk; logistic regression was applied to MASLD, AIP-high, and TyG-high. All models were adjusted *a priori* for age, sex, and BMI, and liver outcomes were also adjusted for HbA1c. Logistic *P*-values were Wald unless otherwise specified. Variance inflation factors remained below 2 in every model, and the Hosmer–Lemeshow goodness-of-fit was reported for the logistic models. Cronbach’s *α* with 95% confidence interval (95% CI) quantified the internal consistency of the MBI subscales and the CD-RISC-10. Prevalence denominators were reported using the available measurement convention. Anthropometric, blood pressure, and psychometric prevalences used set A (*n* = 106). Biochemistry-derived prevalences (including HbA1c-based glycaemic categories, lipid panel, atherogenic surrogate indices, hs-CRP, vitamin D, and Lp(a)) used the assayed denominator within set B (*n* = 103; the haemodialysis participant and two further participants with no biochemistry were excluded). FibroScan-derived prevalences (MASLD and advanced fibrosis) used the FibroScan-tested denominator (*n* = 102). Multivariable regression models used complete-case analysis and were reported with their model-specific *n* in **Table 1** (set A linear FibroScan models *n* = 102; MASLD logistic *n* = 104; set B regression *n* = 104).

**Table 1:** Adjusted multivariable associations of burnout and burnout dimensions with cardiometabolic outcomes.

Model	Exposure	Outcome	Cohort	Estimate (95% CI)	*R*²	*P*
**M1**	EE per +1 unit	LSM (kPa)	Set A FibroScan (*n* = 102)	***β* = 0.061 (0.022, 0.100)**	0.17	**0.002**
**M2**	DP per +1 unit	LSM (kPa)	Set A FibroScan (*n* = 102)	***β* = 0.106 (0.025, 0.187)**	0.14	**0.011**
M3	PA per +1 unit	LSM (kPa)	Set A FibroScan (*n* = 102)	*β* = −0.006 (−0.045, 0.034)	0.08	0.775
M4	EE per +1 unit	CAP (dB/m)	Set A FibroScan (*n* = 102)	*β* = 0.673 (−0.286, 1.631)	0.22	0.167
**M5**	DP per +1 unit	CAP (dB/m)	Set A FibroScan (*n* = 102)	***β* = 2.426 (0.505, 4.346)**	0.26	**0.014**
M6	Burnout (any-dim, 4th)	AIP	Set B regression (*n* = 104)	*β* = 0.087 (−0.011, 0.185)	0.17	0.082
M7	Burnout (any-dim, 4th)	Castelli risk index I	Set B regression (*n* = 104)	*β* = 0.183 (−0.232, 0.598)	0.11	0.383
M8	Burnout (any-dim, 4th)	LAP	Set B regression (*n* = 104)	*β* = 6.20 (−1.77, 14.18)	0.47	0.126
M9	Burnout (any-dim, 4th)	FRS 10-year primary, %	Set B regression (*n* = 104)	*β* = 0.65 (−0.38, 1.67)	0.62	0.212
M10	Burnout (any-dim, 4th)	MASLD	Set A regression (*n* = 104)	OR = 1.58 (0.62, 4.00)	0.29	0.335
M11	EE per +1 unit	MASLD	Set A regression (*n* = 104)	OR = 1.02 (0.97, 1.07)	0.29	0.369
M12	DP per +1 unit	MASLD	Set A regression (*n* = 104)	OR = 1.07 (0.97, 1.18)	0.30	0.172
M13	Burnout (any-dim, 4th)	AIP-high (>0.21)	Set B regression (*n* = 104)	OR = 1.44 (0.52, 3.95)	0.10	0.484
M14	Burnout (any-dim, 4th)	TyG-high (>8.6)	Set B regression (*n* = 104)	OR = 1.06 (0.45, 2.51)	0.13	0.896

Linear regression for continuous outcomes (M1–M9) and logistic regression for binary outcomes (M10–M14). All *P*-values are Wald. Models adjusted for age, sex, and body mass index (BMI); liver outcomes additionally adjusted for glycated haemoglobin (HbA1c). *β* coefficients in original outcome units; OR are adjusted odds ratios. *R*² is adjusted *R*² for linear models, Nagelkerke pseudo-*R*² for logistic models. Variance inflation factors <2 in every model. Bonferroni- and Benjamini–Hochberg-adjusted *q* values for the 14-model family are reported in **Supplementary Table S1**. Hosmer–Lemeshow goodness-of-fit *P*-values (logistic models): M10 *P* = 0.327; M11 *P* = 0.482; M12 *P* = 0.840; M13 *P* = 0.179; M14 *P* = 0.602. All models met the conventional threshold *P* > 0.05. Bold values (models M1, M2, and M5) indicate statistical significance at the uncorrected two-sided threshold P < 0.05.

AIP, atherogenic index of plasma; CAP, controlled attenuation parameter; CI, confidence interval; DP, depersonalisation; EE, emotional exhaustion; FRS, Framingham risk score; LAP, lipid accumulation product; LSM, liver stiffness measurement; MASLD, metabolic dysfunction-associated steatotic liver disease (CAP ≥ 238 dB/m); OR, odds ratio; PA, personal accomplishment; TyG, triglyceride–glucose index.

The 30-variable Mann–Whitney comparison block was Bonferroni-corrected at *α* = 0.0017 (0.05/30). The 14 multivariable regression models in the principal analysis were prespecified by hypothesis (three MBI dimensions × six outcomes, with two binary-burnout exposures included for parameterisation comparison) and were reported without further multiple-testing correction in the primary table. Sensitivity analyses repeated the principal models by excluding participants with diabetes, applying the latent burnout profile classification as a categorical predictor, retaining the haemodialysis participant in biochemistry-based models, and stratifying the Framingham analysis by eGFR ≥90 versus <90 mL/min/1.73 m². All analyses were conducted in IBM SPSS Statistics version 28 with cross-validation in Python (statsmodels and scipy). A two-sided *P <*0.05 was considered statistically significant.

### 2.8 Data corrections

Three data-quality corrections were applied prior to the analysis. One CAP value entered as 3.50 dB/m was corrected to 350 dB/m as a data-entry error, given the physiological CAP range of 100 to 400 dB/m. Two MBI item entries with values exceeding the 0–6 Likert range were treated as typographical errors and corrected to the intended single-digit values. One participant with a single missing DP item had that item imputed using the participant’s within-subscale mean, per the MBI 4th-edition manual rule for two or fewer missing items. Four participants with extensive MBI missingness (more than two items missing on the inventory) were excluded from the psychometric analyses; their cardiometabolic data were retained where available. CCA-IMT data were retained for 101 of 106 MBI-complete participants; no value fell below the 0.20-mm physiological plausibility threshold in the analytic data frame. Age was not recorded numerically for five participants (four not stated, one recorded as a 40 to 50 range). Age-based descriptive statistics use the 101 participants with a recorded numeric age; in the multivariable models, which include age as a covariate, these five participants were assigned the cohort-typical value of 50 years. Mean age was 43.6 ± 10.8 years on complete cases versus 43.9 ± 10.6 years with this assignment, and no model estimate was materially affected. The *post hoc* identification of one haemodialysis participant and the resulting cohort split (set A versus set B) is described in Section 2.2. A full participant-level corrections log is provided in **Supplementary Methods**.

## 3 Results

### 3.1 Cohort characteristics

A total of 110 faculty members completed enrolment. The MBI-complete analytic cohort comprised 106 participants after excluding four cases with substantial missing MBI item missingness. Cardiometabolic data were retained for the four excluded cases where available. The CD-RISC-10–complete cohort comprised 107 participants, and the FibroScan-complete cohort comprised 102 participants. CCA-IMT data were available for 101 of the 106 MBI-complete participants (**Figure 1**). One participant on maintenance haemodialysis was retained for psychometric, anthropometric, blood pressure, and FibroScan analyses but excluded from biochemistry-based and Framingham analyses, yielding a set B cohort of *n* = 105 of whom 103 had assayed biochemistry; descriptive prevalences in this section used the *n* = 103 denominator. Complete-case analysis across covariates further reduced this to *n* = 104 for the multivariable regression models. In the MBI-complete cohort (*n* = 106), median age was 43.0 years (IQR 36.0 to 53.0) with a mean of 43.6 ± 10.8 years, based on the 101 participants with a numerically recorded age; 50.9% were men and 86.8% were married. Mean BMI was 27.1 ± 4.9 kg/m² (median 26.5, IQR 23.9 to 30.2). The anthropometric, vital, biochemical, and imaging characteristics of the cohort are summarised in **Table 2**.

**Table 2:** Baseline characteristics of the MBI-complete cohort (*n* = 106), stratified by any-dimension burnout status (MBI 4th edition).

Variable	All (*n* = 106)	Burnout (*n* = 65)	No burnout (*n* = 41)	*P*
Demographics and anthropometry (set A, *n* = 106)				
Age, years	43.0 (36.0–53.0)	39.0 (35.0–48.0)	47.0 (40.5–54.0)	**0.019**
Male sex, *n* (%)	54 (50.9)	34 (52.3)	20 (48.8)	0.877
BMI, kg/m²	26.5 (23.9–30.2)	26.8 (24.0–30.7)	25.6 (23.9–28.8)	0.392
Overweight (BMI 25–29.9 kg/m²), *n* (%)	34 (32.1)	20 (30.8)	14 (34.1)	0.881
Obesity (BMI ≥ 30 kg/m²), *n* (%)	28 (26.4)	20 (30.8)	8 (19.5)	0.292
Waist circumference, cm	90.0 (82.0–99.0)	91.0 (84.0–101.8)	89.0 (78.8–99.0)	0.296
Waist-to-hip ratio	0.86 (0.77–0.93)	0.88 (0.78–0.94)	0.85 (0.77–0.92)	0.299
Blood pressure				
Systolic BP, mmHg	120.5 (110.8–130.2)	121.0 (112.5–130.2)	120.0 (109.8–131.0)	0.671
Diastolic BP, mmHg	75.0 (68.0–83.0)	76.5 (68.8–82.2)	74.0 (68.0–83.2)	0.495
Prehypertension, *n* (%)	35 (33.0)	22 (33.8)	13 (31.7)	0.987
Hypertension, *n* (%)	16 (15.1)	11 (16.9)	5 (12.2)	0.701
Glycaemic and lipid panel (set B, *n* = 103)				
Fasting glucose, mmol/L	5.48 (5.01–5.95)	5.36 (5.01–5.96)	5.56 (4.97–5.94)	0.884
HbA1c, %	5.2 (5.0–5.6)	5.2 (5.0–5.5)	5.3 (5.0–5.7)	0.792
Total cholesterol, mmol/L	4.16 (3.81–4.79)	4.12 (3.86–4.64)	4.25 (3.74–4.80)	0.858
LDL-C, mmol/L	3.07 (2.49–3.68)	3.08 (2.57–3.58)	2.95 (2.40–3.73)	0.545
HDL-C, mmol/L	1.31 (1.08–1.51)	1.23 (1.04–1.42)	1.38 (1.15–1.71)	**0.010**
Triglycerides, mmol/L	1.21 (0.90–1.76)	1.28 (0.95–1.84)	1.15 (0.84–1.60)	0.130
Atherogenic surrogate indices (set B, *n* = 103)				
Triglyceride–glucose index (TyG)	8.65 (8.26–9.01)	8.68 (8.32–9.05)	8.60 (8.22–8.93)	0.147
TyG high (>8.6), *n* (%)	53 (51.5)	33 (51.6)	20 (48.8)	0.938
Atherogenic index of plasma (AIP)	−0.04 (−0.20 to 0.20)	−0.01 (−0.12 to 0.23)	−0.14 (−0.24 to 0.12)	**0.025**
AIP high (>0.21), *n* (%)	25 (24.3)	17 (26.6)	8 (19.5)	0.553
Castelli risk index I	3.30 (2.74–4.00)	3.41 (2.97–4.23)	3.02 (2.48–3.44)	**0.009**
Castelli risk index II	2.34 (1.78–3.04)	2.50 (2.06–3.10)	2.02 (1.50–2.54)	**0.014**
Lipid accumulation product (LAP)	38.1 (24.7–56.8)	44.6 (28.3–58.2)	33.5 (15.4–46.8)	**0.030**
Visceral adiposity index (VAI)	1.56 (1.01–2.31)	1.74 (1.21–2.53)	1.20 (0.88–2.22)	**0.012**
Hepatic and metabolic imaging (set A FibroScan, *n* = 102)				
LSM, kPa	4.6 (3.7–5.5)	4.8 (3.9–5.8)	4.5 (3.6–5.4)	0.162
CAP, dB/m	244 (206–277)	246 (210–278)	234 (194–274)	0.451
MASLD (CAP ≥ 238 dB/m), *n* (%)	57/102 (55.9)	38/64 (59.4)	19/38 (50%)	0.357
Advanced fibrosis (LSM ≥ 9.7 kPa), *n* (%)	4/102 (3.9)	4/64 (6.3)	0/38 (0.0)	0.294*
Renal and inflammatory (set B, *n* = 103)				
eGFR (CKD-EPI 2021), mL/min/1.73 m²	102.9 (92.4–111.5)	104.9 (93.3–114.5)	100.3 (90.3–108.5)	0.076
hs-CRP, mg/L	1.33 (0.70–3.02)	1.27 (0.70–2.78)	1.73 (0.73–3.65)	0.514
Framingham 10-year CV risk (set B, *n* = 103)				
FRS primary (smoking imputed), %	3.33 (1.45–6.65)	3.12 (1.31–6.47)	4.01 (2.14–6.86)	0.299
Burnout dimensions and resilience (set A, *n* = 106)				
Emotional exhaustion (EE), 0–54	19.0 (13.0–24.8)	22.0 (14.0–32.0)	16.0 (11.0–19.0)	**<0.001**
Depersonalisation (DP), 0–30	5.0 (2.0–8.0)	7.0 (2.0–10.0)	5.0 (2.0–6.0)	**0.022**
Personal accomplishment (PA), 0–48	34.0 (23.0–39.0)	27.0 (19.0–33.0)	39.0 (36.0–42.0)	**<0.001**
CD-RISC-10, 0–40	31.0 (26.0–36.0)	29.0 (24.0–35.0)	33.0 (29.8–36.2)	**0.021**

Continuous variables presented as median (IQR); categorical variables as *n* (%). Group comparisons used Mann–Whitney *U* for continuous and chi-square/Fisher exact for categorical variables. Anthropometric and vital sign rows use set A (*n* = 106). Biochemistry, atherogenic surrogate, eGFR, and Framingham rows use set B (*n* = 103) after exclusion of the haemodialysis participant. Hypertension prevalence uses set A (16 of 106; 15.1%) consistent with the rule in Section 2.2. For participants undergoing FibroScan in set, A the total number was 102 with completed scans.

AIP, atherogenic index of plasma; ALT, alanine transaminase; BMI, body mass index; CAP, controlled attenuation parameter; Castelli I/II, Castelli risk index I/II; CCA-IMT, common carotid intima–media thickness; CD-RISC-10, 10-item Connor-Davidson Resilience Scale; DP, depersonalisation; EE, emotional exhaustion; eGFR, estimated glomerular filtration rate (CKD-EPI 2021); FRS, Framingham risk score; HbA1c, glycated haemoglobin; HDL-C, high-density lipoprotein cholesterol; hs-CRP, high-sensitivity C-reactive protein; IQR, interquartile range; LAP, lipid accumulation product; LDL-C, low-density lipoprotein cholesterol; Lp(a), lipoprotein(a); LSM, liver stiffness measurement; MASLD, metabolic dysfunction-associated steatotic liver disease (CAP ≥ 238 dB/m); PA, personal accomplishment; SBP/DBP, systolic/diastolic blood pressure; TyG, triglyceride–glucose index; VAI, visceral adiposity index; WHR, waist-to-hip ratio.

*Fisher’s exact two-sided test used in preference to chi-square because of expected cell counts <5. All four advanced fibrosis cases were men.Bold values indicate statistical significance at the uncorrected two-sided threshold P < 0.05.

Risk factors were widespread across the cohort. One-third (34 of 106; 32.1%) were overweight, and a further 26.4% (28 of 106) were obese. Hypertension affected 16 of 106 (15.1%) and prehypertension affected an additional 35 (33.0%), placing roughly half the cohort above optimum blood pressure. Among the 103 set B participants with valid biochemistry, prediabetes was present in 17 (16.5%) and overt diabetes in 4 (3.9%). Among the atherogenic surrogates, TyG exceeded 8.6 in 53 of 103 (51.5%), AIP was elevated in 25 of 103 (24.3%), and Castelli I was elevated in 14 of 103 (13.6%). Lp(a) reached borderline elevation (75–124 nmol/L) in 15 of 103 (14.6%) and high elevation (≥125 nmol/L) in 18 of 103 (17.5%); 9 of the 18 high-Lp(a) participants were reported by the laboratory as exceeding the assay’s upper limit of quantification (>240 nmol/L) and were classified as high-Lp(a) for categorical analyses. hs-CRP exceeded 3 mg/L in 27 of 103 (26.2%), and 25-hydroxyvitamin D fell below 30 ng/mL in 50 of 103 (48.5%). Among the 102 FibroScan-tested participants, transient elastography identified MASLD (CAP ≥ 238 dB/m) in 57 (55.9%) and advanced fibrosis (LSM ≥ 9.7 kPa) in 4 (3.9%). Extended biochemistry, lipid surrogate and imaging characteristics are given in **Supplementary Table S2**.

### 3.2 Burnout prevalence and profile distribution

**Figure 2:**
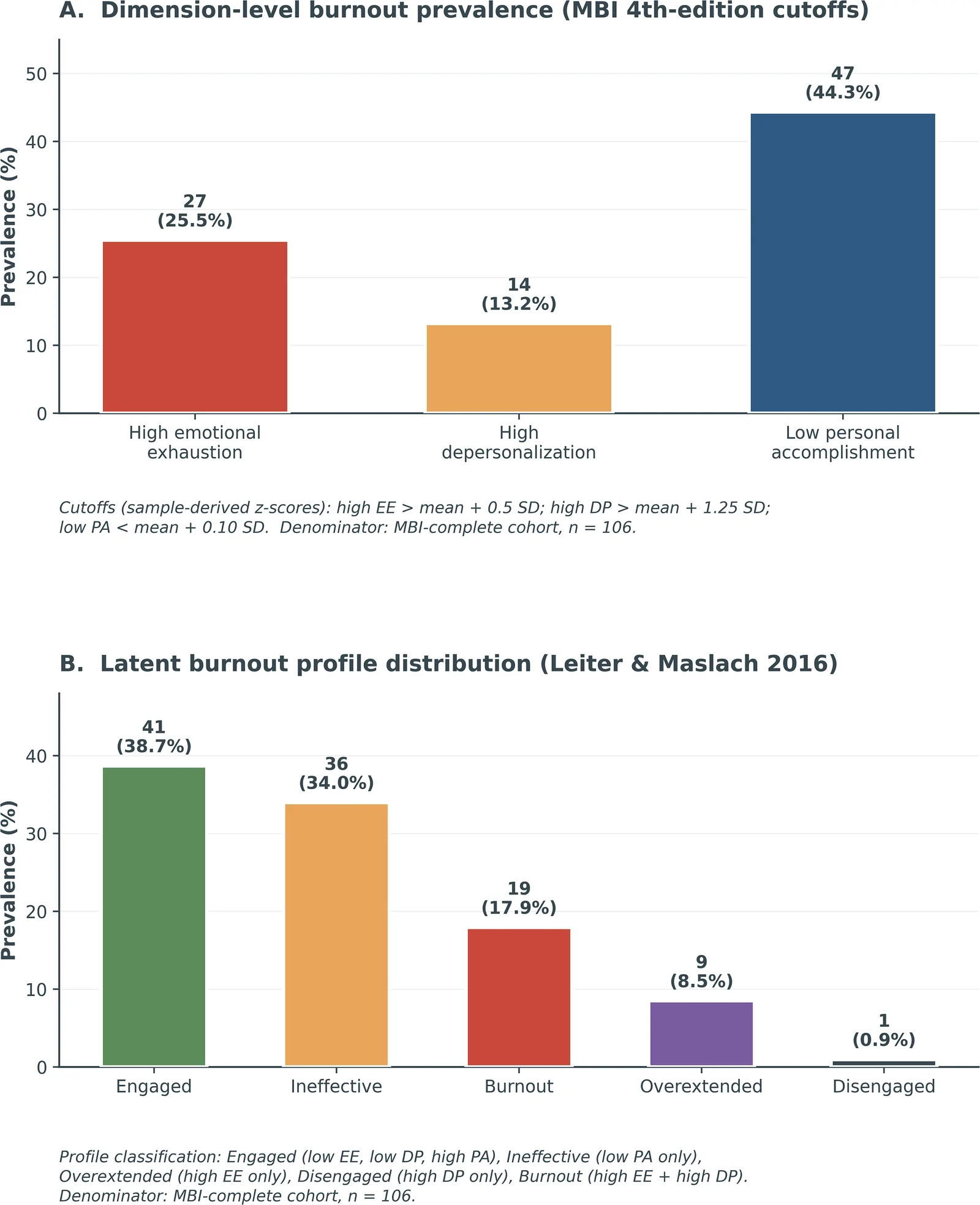
Burnout prevalence and latent profile distribution. MBI-complete cohort, n = 106. **(A)** Dimension-level burnout prevalence under MBI 4th-edition sample-derived *z*-score profile cutoffs [high emotional exhaustion (EE) > mean + 0.5 SD; high depersonalisation (DP) > mean + 1.25 SD; low personal accomplishment (PA) < mean + 0.10 SD]. Counts and percentages: high EE 27 (25.5%), high DP 14 (13.2%), and low PA 47 (44.3%). **(B)** Latent burnout profile distribution per Leiter and Maslach: engaged 41 (38.7%), ineffective 36 (34.0%), burnout 19 (17.9%), overextended 9 (8.5%), and disengaged 1 (0.9%). MBI, Maslach Burnout Inventory.

Within the MBI-complete cohort, any-dimension burnout was present in 65 of 106 participants (61.3%) using the 4th-edition profile cutoffs. Strict three-dimension burnout was present in 4 of 106 (3.8%). At the dimension level under the 4th-edition criteria, 25.5% of the cohort had high emotional exhaustion (27 of 106), 13.2% had high depersonalisation (14 of 106), and 44.3% had low personal accomplishment (47 of 106). The most common latent profile was engaged, observed in 41 participants (38.7%), followed by ineffective in 36 (34.0%), burnout in 19 (17.9%), overextended in 9 (8.5%), and disengaged in 1 (0.9%). Burnout prevalence by dimension and the latent profile distribution are illustrated in **Figure 2**.

Internal consistency was acceptable to good across all instruments: Cronbach’s *α* = 0.85 (95% CI 0.80 to 0.89) for emotional exhaustion, 0.73 (95% CI 0.64 to 0.80) for depersonalisation, and 0.78 (95% CI 0.71 to 0.84) for personal accomplishment. The CD-RISC-10 had Cronbach’s *α* = 0.88 (95% CI 0.84 to 0.91) in the all-available CD-RISC cohort (*n* = 107) and *α* = 0.87 (95% CI 0.82 to 0.90) within the MBI-complete cohort (*n* = 105), with a median CD-RISC-10 score of 31 (IQR 26 to 36), broadly comparable to the original Connor and Davidson development sample (29).

### 3.3 Bivariate associations of burnout with cardiometabolic markers

**Figure 3:**
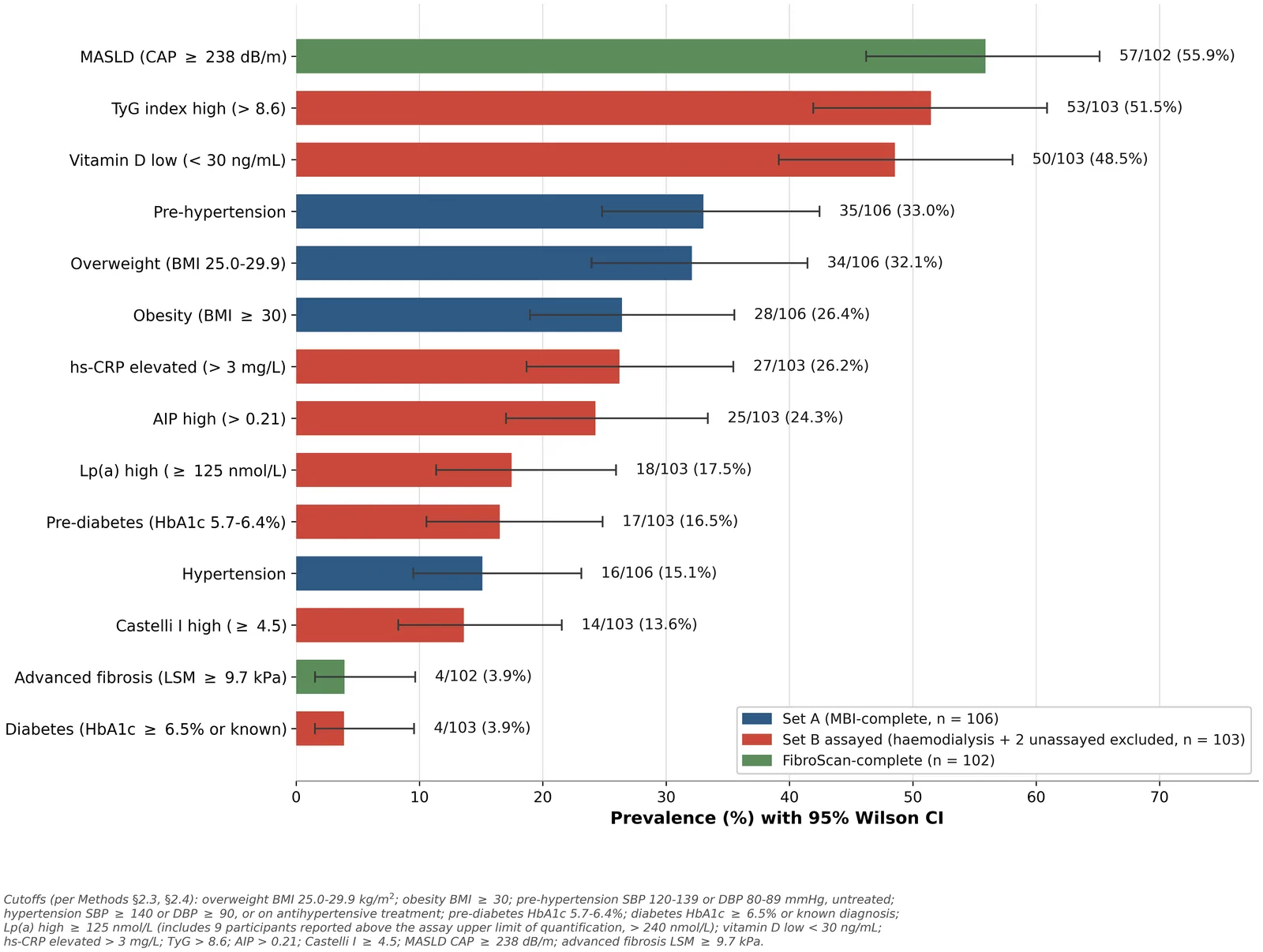
Cardiometabolic risk factor prevalence in the CMHS faculty cohort. Cohort denominators noted on each bar. Set A (MBI-complete, *n* = 106) for anthropometric and blood pressure rows; set B assayed (*n* = 103, after exclusion of the haemodialysis participant and two participants with no biochemistry) for biochemistry-derived rows (TyG, AIP, Castelli I, Lp(a), hs-CRP, vitamin D, prediabetes/diabetes); FibroScan-tested cohort (*n* = 102) for MASLD and advanced fibrosis. Lp(a) high includes nine participants whose laboratory result was reported as exceeding the upper limit of quantification (>240 nmol/L) and is therefore unambiguously above the ≥125-nmol/L high-Lp(a) threshold. Error bars are 95% Wilson confidence intervals. Lp(a), lipoprotein(a); hs-CRP, high-sensitivity C-reactive protein; TyG, triglyceride–glucose index; AIP, atherogenic index of plasma; MASLD, metabolic dysfunction-associated steatotic liver disease; CAP, controlled attenuation parameter; LSM, liver stiffness measurement; SBP/DBP, systolic/diastolic blood pressure.

Because the three MBI subscales define 4th-edition burnout-group membership, between-group differences on these dimensions are mathematically expected and are reported for completeness rather than as empirical findings; the bivariate comparisons in **Table 3** are restricted to variables not used in the burnout classifier. At the raw a = 0.05 level, the burnout group also showed higher Castelli risk index I (3.41 versus 3.02; r = 0.26, P = 0.009), lower HDL-C (median 1.23 versus 1.38 mmol/L; r = 0.25, P = 0.010), higher VAI (1.74 versus 1.20; r = 0.25, P = 0.012), higher Castelli risk index II (2.50 versus 2.02; r = 0.24, P = 0.014), lower median age (39.0 versus 47.0 years; r = 0.23, P = 0.019), lower CD-RISC-10 score (29.0 versus 33.0; r = 0.23, P = 0.021), higher DP score (7.0 versus 5.0; r = 0.22, P = 0.022), higher AIP (−0.01 versus −0.14; r = 0.22, P = 0.025), and higher LAP (44.6 versus 33.5; r = 0.21, P = 0.030). None of these bivariate associations met the Bonferroni-corrected significance threshold for the 30-test family (*α* = 0.0017); the principal multiply-adjusted evidence is reported in the multivariable models in **Table 1**. LSM, CAP, CCA-IMT, BMI, blood pressure, and HbA1c did not differ between burnout groups in unadjusted comparisons (**Table 3**). The full risk-factor prevalence panel is shown in **Figure 3**. Distributions of cardiometabolic markers by burnout status are shown in **Supplementary Figure S1**, the full Spearman correlation matrix in **Supplementary Figure S2**, and the resilience-burnout scatter plots in **Supplementary Figure S3**.

**Table 3:** Mann–Whitney *U* comparisons of 30 cardiometabolic and resilience variables by any-dimension burnout, MBI-complete cohort.

Variable	Burnout median (*n* = 65)	No-burnout median (*n* = 41)	Effect size *r*	*P*	Sig.
Castelli I	3.41	3.02	+0.26	**0.009**	**†**
HDL-C, mmol/L	1.23	1.38	+0.25	**0.010**	**†**
VAI	1.74	1.20	+0.25	**0.012**	**†**
Castelli II	2.50	2.02	+0.24	**0.014**	**†**
Age, years	39.0	47.0	+0.23	**0.019**	**†**
CD-RISC-10 total	29.0	33.0	+0.23	**0.021**	**†**
DP score	7.00	5.00	+0.22	**0.022**	**†**
AIP	−0.01	−0.14	+0.22	**0.025**	**†**
LAP	44.6	33.5	+0.21	**0.030**	**†**
eGFR, mL/min/1.73 m²	104.9	100.3	+0.18	0.076	
Triglycerides, mmol/L	1.28	1.15	+0.15	0.130	
TyG	8.68	8.60	+0.14	0.147	
LSM, kPa	4.75	4.45	+0.14	0.162	
Non-HDL, mmol/L	3.00	2.95	+0.11	0.273	
Waist circumference, cm	91.0	89.0	+0.10	0.296	
Waist-to-hip ratio	0.88	0.85	+0.10	0.299	
FRS primary, %	3.12	4.01	+0.10	0.299	
FRS smoker-equivalent, %	5.28	6.64	+0.10	0.308	
Lp(a), nmol/L	35.1	48.1	+0.09	0.363	
BMI, kg/m²	26.8	25.6	+0.08	0.392	
CAP, dB/m	246.0	234.5	+0.07	0.451	
Diastolic BP, mmHg	76.5	74.0	+0.07	0.495	
hs-CRP, mg/L	1.27	1.73	+0.06	0.514	
LDL-C, mmol/L	3.08	2.95	+0.06	0.545	
Systolic BP, mmHg	121.0	120.0	+0.04	0.671	
CCA-IMT mean, mm	0.50	0.50	+0.03	0.730	
HbA1c, %	5.25	5.25	+0.03	0.792	
ALT, U/L	19.7	20.5	+0.02	0.852	
Total cholesterol, mmol/L	4.12	4.25	+0.02	0.858	
Fasting glucose, mmol/L	5.36	5.56	+0.01	0.884	

Within the MBI-complete cohort (*n* = 106; burnout = 65, no-burnout = 41). Effect size *r* = *Z*/√*N*. Biochemistry-based and Framingham variables used set B (*n* = 103) after exclusion of the haemodialysis participant. CCA-IMT was available for 101 of 106 MBI-complete participants. Significance markers: *P < 0.0017 (Bonferroni-corrected for 30 tests; *α* = 0.0017); †*P* < 0.05 (uncorrected, raw *α*).

AIP, atherogenic index of plasma; ALT, alanine transaminase; Castelli I, Castelli risk index I (total cholesterol/HDL-C); Castelli II, Castelli risk index II (LDL-C/HDL-C); CCA-IMT, common carotid intima–media thickness; CD-RISC-10, 10-item Connor-Davidson Resilience Scale; DP, depersonalisation; eGFR, estimated glomerular filtration rate (CKD-EPI 2021); FRS, Framingham risk score; HDL-C/LDL-C, high-/low-density lipoprotein cholesterol; hs-CRP, high-sensitivity C-reactive protein; LAP, lipid accumulation product; Lp(a), lipoprotein(a); MBI, Maslach Burnout Inventory; TyG, triglyceride–glucose index; VAI, visceral adiposity index. Effect size r is reported as absolute magnitude; the direction of each difference is given by the group medians.Bold values indicate statistical significance at the uncorrected two-sided threshold P < 0.05.

Spearman’s correlation across 24 burnout, resilience, and cardiometabolic variables identified the expected atherogenic cluster structure (TRIG, TyG, AIP, LAP, Castelli I, BMI, and waist circumference were all positively intercorrelated with *ρ* between 0.34 and 0.95) and an age-driven Framingham cluster (FRS was correlated with age at *ρ* = 0.82, with HbA1c at *ρ* = 0.45, and inversely with eGFR at *ρ* = −0.55). Within the burnout block, EE and DP were strongly intercorrelated (*ρ* = 0.63, *P* = 6.3 × 10^-13^) and PA correlated positively with the CD-RISC-10 (*ρ* = 0.31, *P* = 0.001), whereas EE correlated inversely with the CD-RISC-10 (*ρ* = −0.27, *P* = 0.006). The DP × CD-RISC-10 correlation was not statistically significant (*ρ* = −0.17, *P* = 0.077). EE was positively correlated with LSM (*ρ* = 0.23, *P* = 0.020) and DP was positively correlated with CAP (*ρ* = 0.24, *P* = 0.016).

### 3.4 Multivariable models

After adjustment for age, sex, BMI, and HbA1c (for liver outcomes), each one-point increase in EE was associated with a 0.061-kPa increase in LSM (95% CI, 0.022 to 0.100; Wald *P* = 0.002; adjusted *R*² = 0.17), and each one-point increase in DP with a 0.106-kPa increase in LSM (95% CI, 0.025 to 0.187; *P* = 0.011; adjusted *R*² = 0.14) and a 2.43-dB/m increase in CAP (95% CI, 0.51 to 4.35; *P* = 0.014; adjusted *R*² = 0.26). PA was not associated with LSM (*β* = −0.006 kPa, 95% CI −0.045 to 0.034, *P* = 0.775).

**Figure 4:**
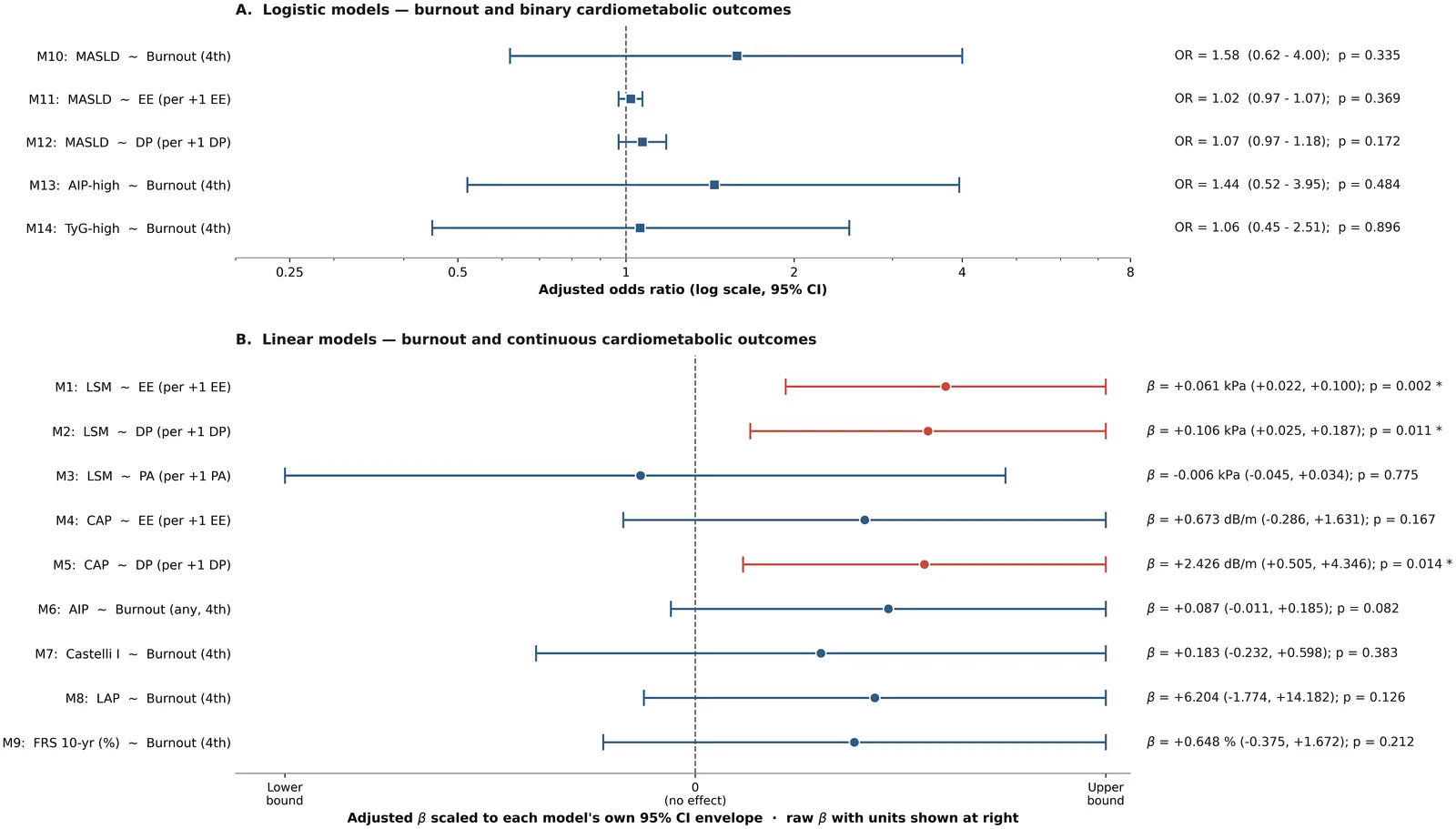
Adjusted associations of burnout and burnout dimensions with cardiometabolic outcomes. Forest plot of all 14 multivariable models reported in **Table 1**. **(A)** Logistic models (M10–M14) for binary outcomes shown as adjusted odds ratios on a log scale; **(B)** linear models (M1–M9) for continuous outcomes shown with *β* coefficients in original outcome units. All models adjusted for age, sex, and BMI; liver outcomes (LSM, CAP, MASLD) additionally adjusted for HbA1c. Coral markers indicate 95% CI excluding the null reference (OR = 1 or *β* = 0); blue markers indicate 95% CI crossing the null. Burnout (4th) = any-dimension burnout under MBI 4th-edition profile cutoffs. EE, emotional exhaustion; DP, depersonalisation; PA, personal accomplishment; LSM, liver stiffness measurement; CAP, controlled attenuation parameter; LAP, lipid accumulation product; AIP, atherogenic index of plasma; MASLD, metabolic dysfunction-associated steatotic liver disease; TyG, triglyceride–glucose index; FRS, Framingham risk score.

The dichotomous-burnout models showed a weaker signal. The adjusted odds ratio for MASLD given any-dimension burnout was 1.58 (95% CI, 0.62–4.00; Wald *P* = 0.335). Continuous-dimension MASLD models gave adjusted ORs of 1.02 per 1-point EE (95% CI, 0.97–1.07; *P* = 0.369) and 1.07 per 1-point DP (95% CI, 0.97–1.18; *P* = 0.172), neither of which were significant. In the biochemistry-derived cohort (*n* = 104), the adjusted burnout-AIP coefficient was close to the threshold but did not cross it (*β* = 0.087, 95% CI, −0.011 to 0.185; *P* = 0.082); adjusted associations with Castelli I (*β* = 0.183, *P* = 0.383), LAP (*β* = 6.20, *P* = 0.126), and FRS (*β* = 0.65, 95% CI, −0.38 to 1.67; *P* = 0.212) likewise did not reach significance. Adjusted ORs for AIP-high and TyG-high were 1.44 (95% CI, 0.52–3.95; *P* = 0.484) and 1.06 (95% CI, 0.45–2.51; *P* = 0.896), respectively. The burnout-Framingham coefficient should be interpreted as a population-mean expected-risk increment rather than a participant-specific risk increment because Framingham scores were computed using sex-stratified imputed smoking probabilities (0.15 for men and 0.02 for women) rather than directly observed smoking status. The full set of adjusted estimates is summarised in **Figure 4** (forest plot) and tabulated in **Table 1**. Bonferroni-corrected and Benjamini–Hochberg-adjusted q values for the 14-model family are given in **Supplementary Table S1**.

### 3.5 Sensitivity analyses

Excluding the four participants with diabetes (HbA1c ≥ 6.5%) did not change the burnout–MASLD adjusted odds ratio meaningfully (OR 1.58, 95% CI 0.62 to 4.07, Wald *P* = 0.341). The latent burnout profile parameterisation produced no significant odds ratios for MASLD versus the engaged reference group. The principal multivariate models reported in **Table 1** already excluded the haemodialysis participant from biochemistry-based and Framingham analyses (set B regression cohort, *n* = 104). A reciprocal sensitivity analysis retaining the haemodialysis participant in biochemistry-based models did not qualitatively change any conclusion: the burnout–MASLD logistic models remained non-significant, and the burnout-Framingham coefficient remained directionally adverse but non-significant, consistent with the haemodialysis participant’s elevated Framingham score being driven by age and treated hypertension rather than by burnout exposure.

The Framingham primary estimate was, by construction, almost perfectly rank-correlated with both the non-smoker-equivalent and smoker-equivalent bounds (Spearman’s *ρ* = 0.999 in both comparisons) because the smoking input was applied as a population-level constant within each sex stratum and therefore did not alter within-cohort rank order. The clinically meaningful sensitivity exhibited is the risk category shift: 6 of 103 (5.8%) participants shifted category between the primary and non-smoker-equivalent estimates, whilst 47 of 103 (45.6%) shifted between the primary and smoker-equivalent estimates. The asymmetry reflects the strong leverage of any non-zero smoking input in this age range and supports the interpretation of the surrogate atherogenic indices in parallel with Framingham. In the eGFR-stratified Framingham sensitivity analysis restricted to participants with eGFR of 30 mL/min/1.73 m² or greater (*n* = 103 after exclusion of the haemodialysis participant and two further cases with eGFR data unavailable), Spearman’s *ρ* between FRS and eGFR was −0.543 overall (*P* = 3.1 × 10^-9^), −0.643 within the burnout-present subgroup (*n* = 63, *P* = 1.3 × 10^-8^), and −0.265 within the burnout-absent subgroup (*n* = 40, *P* = 0.099). The apparent steeper inverse association within the burnout-present subgroup is exploratory and does not constitute a formal interaction test. A descriptive scatter plot of Framingham 10-year risk against eGFR by burnout status is shown in **Supplementary Figure S4**.

## 4 Discussion

To the best of our knowledge, this is the first integrated assessment of cardiometabolic phenotype, burnout, and resilience in an academic medical faculty in a Gulf setting. Three principal findings emerged. First, both cardiometabolic risk burden and burnout prevalence in the CMHS faculty are substantial, with 61.3% of participants meeting any-dimension burnout under MBI 4th-edition criteria and over half carrying MASLD by transient elastography. Second, after adjusting for age, sex, and BMI, emotional exhaustion and depersonalisation were independently associated with measurable hepatic phenotype (LSM and CAP), whereas personal accomplishment was not. Third, despite higher unadjusted atherogenic dyslipidaemia in the burnout group (lower HDL-C and higher AIP, Castelli risk indices, VAI, and LAP), the dichotomous burnout−MASLD association did not reach statistical significance after multivariate adjustment, consistent with a dimension-specific rather than a categorical pattern of burnout–cardiometabolic association observed in this cohort.

The 61.3% any-dimension burnout prevalence in the CMHS faculty is consistent with the upper end of physician burnout estimates from the recent literature, which range from 40% to 70% depending on instrument version and case definition (8, 9, 15, 31, 32). The dimension-specific finding that emotional exhaustion and depersonalisation, but not reduced personal accomplishment, were independently associated with hepatic phenotype is biologically plausible. Both emotional exhaustion and depersonalisation have been linked in mechanistic studies to dysregulated cortisol secretion, blunted interleukin-6 stress reactivity, and coronary microvascular dysfunction in physician cohorts (9–12, 33), and these neuroendocrine and inflammatory pathways converge on hepatic insulin resistance and *de novo* lipogenesis, the central physiologic drivers of MASLD (34). Personal accomplishment is conceptually distinct, reflecting a self-evaluative achievement construct that may be more weakly coupled to physiologic stress activation (27, 28). The observation that PA and CD-RISC-10 resilience were positively correlated (*ρ* = 0.31) whilst EE and CD-RISC-10 were inversely correlated (*ρ* = −0.27) further supports the case that the protective behavioural mechanisms captured by resilience map preferentially onto the personal accomplishment dimension rather than the load and disengagement dimensions of burnout.

The high prevalence of MASLD (55.9%) in this academic faculty cohort exceeds estimates for community-dwelling healthy adults in South Asia and the Gulf, where MASLD prevalence on FibroScan typically ranges from 21% to 44% (5, 6, 35, 36). This excess is best understood in the context of the substantial baseline cardiometabolic risk burden documented in this cohort. Half of the participants had elevated TyG, almost a quarter had elevated AIP, and over a quarter were obese. The atherogenic dyslipidaemia phenotype documented here, with lower HDL-C and elevated Castelli I, AIP, LAP, and VAI in the burnout group, mirrors the Weqaya population-level Emirati profile, in which obesity (35%), diabetes (18%), dyslipidaemia (44%), and hypertension (23%) cluster together in the working-age national population (1, 37). The first international report of MASLD by FibroScan in Emirati medical students reported a prevalence of approximately 21% (6), whereas the Saudi temporal-trends study in young adults found that hepatic steatosis prevalence is rising rapidly in this region (7).

The four advanced-fibrosis cases (LSM ≥ 9.7 kPa) occurred entirely within the burnout group (4 of 64 burnout-positive participants versus 0 of 38 burnout-negative; Fisher’s exact *P* = 0.29), and all four were men. This distribution is consistent with the dimension-specific continuous association between EE/DP and LSM (M1, M2), but the categorical comparison is underpowered (≈4 events in 102 participants) and should not be overinterpreted. A larger prospective cohort would be required to test whether advanced fibrosis is selectively enriched in the burnout-positive faculty.

Of the 103 set B participants with assayed Lp(a), 18 (17.5%) had high Lp(a) (≥125 nmol/L), of whom 9 were reported by the laboratory as exceeding the assay’s upper limit of quantification (>240 nmol/L). This prevalence exceeds the approximately 10%–15% range commonly cited for unselected populations and represents a genetically determined residual cardiovascular risk that is independent of the burnout exposure but clinically meaningful in a Gulf academic cohort, given that high Lp(a) is associated with accelerated atherosclerotic cardiovascular disease and remains a treatment-gap target.

The Framingham 10-year cardiovascular risk in this cohort showed only a non-significant burnout-related increment of 0.65 percentage points after adjustment, despite the substantial unadjusted atherogenic dyslipidaemia in the burnout group. By contrast, a larger health-worker cohort applying the same Framingham tool reported burnout as an independent predictor of high 10-year cardiovascular risk (38), a difference attributable in part to that study’s larger sample and directly measured smoking status. Three methodological factors are relevant. First, the Framingham general CVD equation has known calibration limitations in Gulf and South Asian populations, with multiple validation studies reporting overestimation of observed event rates by 30%–100% in Middle Eastern cohorts (24, 33). The very strong age dependence of the Framingham score (Spearman *ρ* = 0.82 with age in this cohort) absorbs much of the variance attributable to other risk factors. Second, smoking prevalence could not be measured directly and was imputed at sex-stratified UAE national values; the score should therefore be interpreted as a population-mean expected-risk profile rather than a participant-specific predicted risk. Third, the smoker-equivalent sensitivity bound produced a 45.6% risk category shift versus the primary, demonstrating that the Framingham score is highly sensitive to smoking input in this age range. The atherogenic surrogate indices (AIP, Castelli I, LAP, VAI), which are smoking-independent and have growing validation in Gulf cardiometabolic populations (21, 22), may therefore offer a more sensitive readout of burnout-associated atherogenic phenotype in academic faculty than Framingham score alone.

Resilience as measured by CD-RISC-10 showed the expected inverse relationship with emotional exhaustion (*ρ* = −0.27) and a positive relationship with personal accomplishment (*ρ* = 0.31), consistent with prior Iranian and Tehrani nurse cohorts using the CD-RISC-25 or CD-RISC-10 instruments (16–18). The cohort median CD-RISC-10 score of 31 falls within the upper range reported for Middle Eastern healthcare worker samples and is broadly comparable to the original Connor and Davidson development sample (29). A Middle Eastern academic faculty normative dataset for CD-RISC-10 has not been published; the present cohort therefore contributes one of the first Gulf region academic faculty resilience benchmarks for future comparison.

This study has several limitations. First, the cross-sectional design precludes causal inference, and the directionality of the burnout–hepatic phenotype association cannot be determined. Faculty with elevated cardiometabolic risk may experience greater fatigue and emotional exhaustion as a consequence of their metabolic phenotype, rather than vice versa. Second, the single-centre design limits generalisability beyond academic medical faculty in the Gulf region. Third, smoking status was not captured at the screening visit, so the Framingham score was computed using sex-stratified national smoking probabilities (0.15 for men and 0.02 for women). This sex-level imputation is the principal limitation of the Framingham analysis, and the resulting coefficient (M9) should be read as a population-mean expected-risk profile rather than a participant-specific estimate. We report non-smoker- and smoker-equivalent sensitivity bounds, and within-cohort rank order is preserved across all three estimates (Spearman *ρ* = 0.999). The smoker-equivalent bound nonetheless reclassified 45.6% of participants across risk categories, which quantifies how sensitive the estimate is to this single unmeasured variable. Any burnout-related Framingham increment is therefore best regarded as exploratory and interpreted alongside the smoking-independent atherogenic surrogate indices (AIP, Castelli I, LAP, VAI), which provide a more robust cardiovascular-phenotype readout in this study. Future faculty-screening protocols should capture individual smoking status directly and, ideally, apply a Gulf-recalibrated risk equation, so that cardiovascular risk is estimated at the participant level rather than inferred from population probabilities. Fourth, the modest sample size (*n* = 106 MBI-complete) limited power for the categorical burnout–MASLD logistic models; the dimension-specific linear models retained adequate power because they used continuous predictors. Fifth, the Framingham equation has documented calibration limitations in Middle Eastern populations (33), and a Gulf-recalibrated risk score would be preferable for future work. Sixth, the four MBI-excluded participants and three CD-RISC-excluded participants represent a 6% loss of psychometric data; cardiometabolic variables were retained where available to minimise this loss. Seventh, the cohort included one participant with established renal failure on maintenance haemodialysis, identified *post hoc* through an outlying creatinine value. This participant was retained for psychometric and imaging analyses but excluded from biochemistry-based and Framingham analyses, on the grounds that pharmacotherapy and renal replacement therapy effects distort the lipid and inflammatory profile away from physiological cardiometabolic phenotype. Eighth, the bivariate Mann–Whitney comparisons in **Table 3** did not survive Bonferroni correction for the 30-variable comparison family. The principal evidence for the burnout–cardiometabolic association therefore rests on the prespecified dimension-level multivariate models in **Table 1**, of which the EE → LSM model (M1) was the single result surviving both Bonferroni and Benjamini–Hochberg correction at *α* = 0.05.

Despite these limitations, this study provides the first integrated burnout, resilience, and objective cardiometabolic phenotype dataset from a Gulf academic medical faculty cohort, with transient elastography, atherogenic surrogate indices, and common carotid intima–media thickness measured in the same individuals. The dimension-specific findings offer a working hypothesis for future prospective and interventional work on physician wellness in the region.

Four clinical and occupational implications follow. First, Gulf academic medical wellness programmes should add routine cardiometabolic phenotyping, anthropometry, blood pressure, fasting lipid and glycaemic panel, atherogenic surrogate indices, and transient elastography where available, to existing burnout and resilience screening, because the two clustered together in this cohort. Second, screening should report the three MBI dimensions separately rather than collapsing them into a global score because the burnout–cardiometabolic signal in our data is dimension-specific. Third, given the high MASLD prevalence and the responsiveness of liver stiffness to weight reduction and metabolic intervention, FibroScan deserves a place in faculty wellness pathways wherever the modality is accessible. Fourth, from an occupational health standpoint, the co-clustering of burnout with adverse cardiometabolic phenotype supports embedding cardiometabolic screening within existing institutional faculty wellness and periodic health examination programmes rather than running the two as separate initiatives, an approach that shares laboratory and imaging resources and may improve uptake amongst time-constrained faculty. Because emotional exhaustion and depersonalisation were the dimensions coupled to measurable liver phenotype, occupational health services may reasonably prioritise workload and disengagement drivers, alongside conventional metabolic risk factor management, when designing interventions for this workforce. These implications are hypothesis-generating and require confirmation in prospective and multicentre faculty cohorts before they inform formal screening policy.

## 5 Conclusions

This Gulf academic faculty cohort carried substantial cardiometabolic risk that co-clustered with specific burnout dimensions. After adjustment for age, sex, BMI, and HbA1c, emotional exhaustion and depersonalisation were associated with measurable liver phenotype (LSM and CAP), whereas reduced personal accomplishment was not; resilience showed the expected inverse correlation with emotional exhaustion and positive correlation with personal accomplishment. Two features distinguish this work from the prior literature. First, to our knowledge, it is the first cohort to pair objective transient elastography, carotid, and atherogenic surrogate phenotyping with dimension-resolved burnout and resilience measurement in a Gulf academic medical faculty, whereas earlier studies relied on self-reported cardiometabolic outcomes or a single global burnout score. Second, the association with hepatic phenotype was confined to the emotional exhaustion and depersonalisation dimensions rather than to a binary burnout label, which argues for dimension-specific rather than categorical burnout reporting in wellness screening. Because the design is cross-sectional, these relationships are associative and do not establish direction of causation. The findings nonetheless support integrated cardiometabolic, hepatic, and burnout screening, with dimension-specific reporting, in academic medical faculty wellness programmes across the United Arab Emirates and the wider Gulf region, and they define testable hypotheses for future prospective and interventional studies.

## Data Availability

The raw data supporting the conclusions of this article will be made available by the authors, without undue reservation.
