# Supplementary material for: Burnout, resilience, and cardiometabolic phenotype in an academic medical faculty: an integrated cross-sectional assessment from the United Arab Emirates
Source: Front Endocrinol (Lausanne). 2026 Aug 17;17:1913703. doi: 10.3389/fendo.2026.1913703 (PMC13526673; doi:10.3389/fendo.2026.1913703)
Supplement: Supplementary file 1 [file SupplementaryFile1.docx]

**Supplementary Material**

**Contents**

• Supplementary Methods — participant-level data-corrections log (S-M.1 to S-M.7)

• Supplementary Table S1 — multiple-testing corrections for the 14 multivariable models in Table 1

• Supplementary Table S2 — extended cohort characteristics

• Supplementary Figures S1 to S4 — legends below; figures supplied as separate high-resolution files

**Supplementary Methods. Participant-level data-corrections log**

Each data-quality correction applied before analysis is documented below at participant level so that readers can trace its effect on derived variables.

**S-M.1 CAP unit-conversion correction**

One participant controlled attenuation parameter (CAP) value was originally entered as 3.50 dB/m. The physiological CAP range for transient elastography is approximately 100 to 400 dB/m. The value was treated as a decimal-place data-entry error and corrected to 350 dB/m. The correction affects one row in the CAP descriptive analysis and the MASLD classification, since the corrected value exceeds the MASLD threshold of 238 dB/m. The participant’s full FibroScan record (LSM, interquartile-range-to-median ratio, probe selection) was reviewed and was internally consistent with a CAP of 350 dB/m.

**S-M.2 MBI item Likert-range corrections**

Two MBI-HSS-MP item entries were recorded outside the permitted 0 to 6 Likert range: one entry of 66, corrected to 6, and one entry of 7, corrected to 0. Both were treated as typographical data-entry errors. Each corrected value was checked against the participant’s adjacent items for direction-of-error consistency, and both produce subscale totals consistent with the participant’s overall response pattern.

**S-M.3 MBI single-item imputation**

One participant had a single missing depersonalization item. The item was imputed using that participant’s within-subscale mean, per the MBI 4th-edition manual rule for two or fewer missing items in a subscale. The imputed value was 4.4, within the observed range of the participant’s other depersonalization items. This is the only MBI imputation in the dataset; no other missing items were imputed.

**S-M.4 MBI exclusions for extensive missingness**

Four participants had more than two missing items on the inventory and were excluded from psychometric analyses per the MBI 4th-edition manual cutoff. The excluded serial numbers are 48, 65, 108 and 109. Cardiometabolic data for these four participants were retained where available.

**S-M.5 CCA-IMT availability**

Common carotid intima-media thickness was recorded for 101 of the 106 MBI-complete participants. No value fell below the 0.20 mm physiological-plausibility threshold. The five missing measurements arose from operator availability during the screening week and are not informative on either burnout status or cardiometabolic phenotype.

**S-M.6 Post hoc identification of the haemodialysis participant**

One participant was identified *post hoc* through an outlying serum creatinine of 990 µmol/L and an estimated glomerular filtration rate of approximately 5 mL/min/1.73 m². Chart review confirmed maintenance haemodialysis for end-stage renal failure. The participant’s MBI-HSS-MP, CD-RISC-10, anthropometric, blood-pressure and FibroScan records were physiologically plausible and consistent with the rest of the cohort, and the participant was therefore retained in Set A for those analyses. The lipid panel and inflammatory markers reflected pharmacotherapy and renal-replacement-therapy effects rather than physiological cardiometabolic phenotype, and the participant was excluded from all biochemistry-based analyses (Set B), including the atherogenic surrogate indices and the Framingham score. This single *post hoc* exclusion drives the Set A versus Set B cohort split used throughout the manuscript. Two further Set B participants had no blood sample assayed, giving the available-measurement denominator of n = 103 used for all biochemistry-derived descriptive statistics.

**S-M.7 Age recording and handling**

Age was not recorded numerically for five participants: four were recorded as not stated and one as a 40 to 50 range. Age-based descriptive statistics use the 101 participants with a recorded numeric age. In the multivariable models, which include age as a covariate throughout, these five participants were assigned the cohort-typical value of 50 years so that they were not lost to complete-case filtering. Mean age was 43.6 ± 10.8 years on complete cases and 43.9 ± 10.6 years with this assignment; no model estimate was materially affected.

**Supplementary Table S1. Multiple-testing corrections for the 14 pre-specified multivariable models in Table 1**

Effect estimates and 95% confidence intervals are from the IBM SPSS Statistics version 28 output, cross-validated in Python. Cohort denominators: M1 to M5 (FibroScan-based linear models), n = 102, Set A FibroScan-complete; M6 to M9 and M13 to M14 (biochemistry, Framingham and binary biochemistry outcomes), n = 104, Set B regression cohort; M10 to M12 (MASLD logistic), n = 104, Set A complete-case across age, sex, BMI and HbA1c. Raw *P* is the Wald *P* for logistic models and the t-test *P* for linear models. Bonferroni *q* = min(raw *P* × m, 1) with m = 14. Benjamini-Hochberg *q* (false discovery rate): raw *P* ranked ascending, *q* = raw *P* × m / rank, with bottom-up monotonicity applied.

| **Model** | **Outcome** | **Predictor** | **Cohort** | **Effect (95% CI)** | **Raw P** | **Bonferroni q** | **BH q** |
| --- | --- | --- | --- | --- | --- | --- | --- |
| **M1** | Liver stiffness (LSM, kPa) | EE per +1 unit | Set A (n = 102) | β = 0.061 (0.022, 0.100) | **0.002** | **0.035*** | **0.035*** |
| **M2** | Liver stiffness (LSM, kPa) | DP per +1 unit | Set A (n = 102) | β = 0.106 (0.025, 0.187) | **0.011** | 0.158 | **0.065†** |
| M3 | Liver stiffness (LSM, kPa) | PA per +1 unit | Set A (n = 102) | β = −0.006 (−0.045, 0.034) | 0.775 | 1.000 | 0.835 |
| M4 | CAP (dB/m) | EE per +1 unit | Set A (n = 102) | β = 0.673 (−0.286, 1.631) | 0.167 | 1.000 | 0.345 |
| **M5** | CAP (dB/m) | DP per +1 unit | Set A (n = 102) | β = 2.426 (0.505, 4.346) | **0.014** | 0.194 | **0.065†** |
| M6 | Atherogenic index of plasma | Burnout (any-dim, 4th) | Set B (n = 104) | β = 0.087 (−0.011, 0.185) | 0.082 | 1.000 | 0.288 |
| M7 | Castelli risk index I | Burnout (any-dim, 4th) | Set B (n = 104) | β = 0.183 (−0.232, 0.598) | 0.383 | 1.000 | 0.488 |
| M8 | Lipid accumulation product | Burnout (any-dim, 4th) | Set B (n = 104) | β = 6.204 (−1.774, 14.182) | 0.126 | 1.000 | 0.345 |
| M9 | FRS 10-year primary (%) | Burnout (any-dim, 4th) | Set B (n = 104) | β = 0.648 (−0.375, 1.672) | 0.212 | 1.000 | 0.371 |
| M10 | MASLD | Burnout (any-dim, 4th) | Set A (n = 104) | OR = 1.58 (0.62, 4.00) | 0.335 | 1.000 | 0.488 |
| M11 | MASLD | EE per +1 unit | Set A (n = 104) | OR = 1.02 (0.97, 1.07) | 0.369 | 1.000 | 0.488 |
| M12 | MASLD | DP per +1 unit | Set A (n = 104) | OR = 1.07 (0.97, 1.18) | 0.172 | 1.000 | 0.345 |
| M13 | High AIP (> 0.21) | Burnout (any-dim, 4th) | Set B (n = 104) | OR = 1.44 (0.52, 3.95) | 0.484 | 1.000 | 0.564 |
| M14 | High TyG (> 8.6) | Burnout (any-dim, 4th) | Set B (n = 104) | OR = 1.06 (0.45, 2.51) | 0.896 | 1.000 | 0.896 |

** Significant at α = 0.05 after correction. † Close to but not crossing the 5% false discovery rate threshold (BH* ***q*** *< 0.10).*

**Interpretation.** Only M1 (emotional exhaustion → liver stiffness) survives both Bonferroni and Benjamini-Hochberg correction at α = 0.05. M2 (depersonalization → LSM) and M5 (depersonalization → CAP) reach nominal significance (raw *P* ≤ 0.05) with Benjamini-Hochberg *q* = 0.065, close to but not crossing the 5% false discovery rate threshold. No other adjusted association survives multiple-testing correction.

**Reference for the false discovery rate method:** Benjamini Y, Hochberg Y. Controlling the false discovery rate: a practical and powerful approach to multiple testing. *J R Stat Soc Series B Stat Methodol*. 1995;57(1):289–300.

**Supplementary Table S2. Extended cohort characteristics — biochemistry, lipid surrogates and imaging**

Continuous variables are presented as median (interquartile range) and mean ± SD; categorical variables as n (%) with 95% Wilson confidence interval. Set A is the MBI-complete cohort (n = 106) with the haemodialysis participant retained. Set B assayed is the biochemistry-derived cohort (n = 103) after exclusion of the haemodialysis participant and two further participants with no blood sample assayed, consistent with the available-measurement convention stated in Section 2.7.

| **Variable** | **Unit / criterion** | **Cohort (n)** | **Summary** |
| --- | --- | --- | --- |
| **A. Extended biochemistry (Set B assayed, n = 103)** | | | |
| Fasting glucose | *mmol/L* | Set B assayed (103) | 5.48 (5.01–5.95); mean 5.64 ± 0.96 |
| Non-HDL cholesterol | *mmol/L* | Set B assayed (103) | 2.97 (2.46–3.33); mean 2.98 ± 0.76 |
| Alanine transaminase (ALT) | *U/L* | Set B assayed (103) | 19.7 (16.1–24.6); mean 21.8 ± 7.3 |
| Lipoprotein(a) | *nmol/L* | Set B assayed (94) | 42 (20–76); mean 56 ± 50 |
| Lp(a) borderline | *75–124 nmol/L* | Set B assayed (103) | 15 (14.6%); 95% CI 9.0–22.6% |
| Lp(a) high | *≥ 125 nmol/L* | Set B assayed (103) | **18 (17.5%); 95% CI 11.3–25.9%** |
| High-sensitivity CRP | *mg/L* | Set B assayed (103) | 1.33 (0.70–3.02); mean 2.84 ± 3.65 |
| hs-CRP elevated | *> 3 mg/L* | Set B assayed (103) | 27 (26.2%); 95% CI 18.7–35.5% |
| 25-hydroxyvitamin D | *ng/mL* | Set B assayed (103) | 30.3 (23.3–40.2); mean 32.9 ± 14.5 |
| Vitamin D insufficiency or deficiency | *< 30 ng/mL* | Set B assayed (103) | 50 (48.5%); 95% CI 39.1–58.1% |
| eGFR (CKD-EPI 2021) | *mL/min/1.73 m²* | Set B assayed (103) | 102.9 (92.4–111.5); mean 101.5 ± 13.8 |
| **B. Additional atherogenic surrogates (Set B assayed, n = 103)** | | | |
| Castelli risk index II | *LDL-C / HDL-C* | Set B assayed (103) | 2.34 (1.78–3.04); mean 2.53 ± 1.05 |
| Visceral adiposity index (VAI) | *—* | Set B assayed (103) | 1.56 (1.01–2.31); mean 1.87 ± 1.22 |
| **C. Framingham 10-year cardiovascular risk (Set B assayed, n = 103)** | | | |
| FRS 10-year primary (smoking imputed) | *%* | Set B assayed (103) | 3.33 (1.45–6.65); mean 4.59 ± 4.05 |
| FRS 10-year non-smoker equivalent | *%* | Set B assayed (103) | 3.22 (1.31–6.14); mean 4.28 ± 3.68 |
| FRS 10-year smoker equivalent | *%* | Set B assayed (103) | 5.73 (2.51–11.12); mean 7.69 ± 6.65 |
| FRS category: low | *< 5%* | Set B assayed (103) | 69 (67.0%); 95% CI 57.4–75.3% |
| FRS category: borderline | *5 to < 7.5%* | Set B assayed (103) | 16 (15.5%); 95% CI 9.8–23.8% |
| FRS category: intermediate | *7.5 to < 20%* | Set B assayed (103) | 17 (16.5%); 95% CI 10.6–24.9% |
| FRS category: high | *≥ 20%* | Set B assayed (103) | 1 (1.0%); 95% CI 0.2–5.3% |
| **D. Blood-pressure categories (Set A, n = 106)** | | | |
| Pre-hypertension | *SBP 120–139 or DBP 80–89, untreated* | Set A (106) | 35 (33.0%); 95% CI 24.8–42.4% |
| Hypertension | *SBP ≥ 140 or DBP ≥ 90, or treated* | Set A (106) | 16 (15.1%); 95% CI 9.5–23.1% |
| **E. Hepatic phenotype (Set A, FibroScan-complete, n = 102)** | | | |
| Advanced fibrosis | *LSM ≥ 9.7 kPa* | FibroScan (102) | 4 (3.9%); 95% CI 1.5–9.7% |
| **F. Subclinical atherosclerosis (Set A, CCA-IMT measured, n = 101)** | | | |
| Mean common carotid IMT | *mm* | Set A (101) | 0.50 (0.40–0.60); mean 0.49 ± 0.13 |

**Lipoprotein(a) note.** Nine participants had an Lp(a) result reported by the laboratory above the assay upper limit of quantification (> 240 nmol/L). Consistent with Section 2.3, these were counted within the ≥ 125 nmol/L high-Lp(a) category for the categorical rows but excluded from the continuous distribution summary, which therefore uses n = 94.

**Abbreviations:** *ALT, alanine transaminase; CCA-IMT, common carotid intima-media thickness; CKD-EPI, Chronic Kidney Disease Epidemiology Collaboration equation; CRP, C-reactive protein; DBP, diastolic blood pressure; eGFR, estimated glomerular filtration rate; FRS, Framingham risk score; HDL-C, high-density lipoprotein cholesterol; LDL-C, low-density lipoprotein cholesterol; Lp(a), lipoprotein(a); LSM, liver stiffness measurement; SBP, systolic blood pressure; VAI, visceral adiposity index.*

**Supplementary Figure legends**

**Supplementary Figure S1. Cardiometabolic markers by burnout status.**

*Six-panel boxplot comparison of controlled attenuation parameter (CAP, dB/m), liver stiffness measurement (LSM, kPa), mean common carotid intima-media thickness (CCA-IMT, mm), atherogenic index of plasma (AIP), Castelli risk index I and lipid accumulation product (LAP) by any-dimension burnout status under Maslach Burnout Inventory (MBI) 4th-edition cutoffs. MBI-complete cohort, n = 106 (burnout 65, no burnout 41); biochemistry-derived panels use the Set B assayed cohort (n = 103). Mann-Whitney P values are shown in each panel. The between-group differences reaching the uncorrected threshold (Castelli I P = 0.009; AIP P = 0.025; LAP P = 0.030) did not survive Bonferroni correction for the 30-variable comparison family (α = 0.0017) and are reported descriptively.*

**Supplementary Figure S2. Spearman correlation matrix across burnout, resilience and cardiometabolic variables.**

*Lower-triangular heatmap of Spearman ρ across 24 variables in the MBI-complete cohort (n = 106). Cell values are rounded to two decimals; asterisks denote uncorrected P < 0.05. The expected atherogenic cluster (triglyceride-glucose index, AIP, LAP, Castelli risk index I, body mass index, waist circumference) and the age-driven Framingham cluster are both visible. Within the burnout block, emotional exhaustion (EE) and depersonalization (DP) are strongly intercorrelated (ρ = 0.63) and personal accomplishment (PA) correlates positively with the 10-item Connor-Davidson Resilience Scale (CD-RISC-10). EE correlates positively with liver stiffness and DP with controlled attenuation parameter.*

**Supplementary Figure S3. Resilience and burnout dimensions.**

*MBI-complete cohort with an available CD-RISC-10 score (n = 105). Three-panel scatter plot of CD-RISC-10 total score against emotional exhaustion (EE, left), depersonalization (DP, middle) and personal accomplishment (PA, right). Each panel shows a linear regression line with a shaded 95% confidence band on the fitted mean; Spearman ρ tests the rank-order monotonic association. ρ = −0.27 (P = 0.006) for EE; ρ = −0.17 (P = 0.077) for DP; and ρ = 0.31 (P = 0.001) for PA. CD-RISC-10, 10-item Connor-Davidson Resilience Scale; MBI, Maslach Burnout Inventory.*

**Supplementary Figure S4. Framingham 10-year cardiovascular risk versus eGFR by burnout status.**

*Scatter plot of the Framingham 10-year primary cardiovascular risk score against estimated glomerular filtration rate (eGFR) under the CKD-EPI 2021 equation, stratified by any-dimension burnout (MBI 4th edition). Restricted to participants with eGFR ≥ 30 mL/min/1.73 m² (n = 103 after exclusion of the haemodialysis participant and two further cases with eGFR data unavailable). Spearman ρ = −0.543 overall (P < 0.001); ρ = −0.643 within the burnout-present subgroup (n = 63, P < 0.001); ρ = −0.265 within the burnout-absent subgroup (n = 40, P = 0.099). The apparently steeper inverse association in the burnout-present subgroup is exploratory and does not constitute a formal interaction test. Solid lines show the linear regression fit per subgroup with a shaded 95% confidence band. The dashed vertical line marks eGFR = 90 mL/min/1.73 m².*
